# Supplementary material for: Multi‐dataset identification of innovative feature genes and molecular mechanisms in keratoconus
Source: J Cell Mol Med. 2024 Sep 19;28(18):e70079. doi: 10.1111/jcmm.70079 (PMC11412914; doi:10.1111/jcmm.70079)
Supplement: Supplementary file 2 — Material S2. [file JCMM-28-e70079-s002.zip › 7.GSEA analysis/3.DBNDD1/KEGG_EPITHELIAL_CELL_SIGNALING_IN_HELICOBACTER_PYLORI_INFECTION.html]

Details for gene set KEGG\_EPITHELIAL\_CELL\_SIGNALING\_IN\_HELICOBACTER\_PYLORI\_INFECTION[GSEA]

|  || Dataset | DBNDD1.group.cls#h\_versus\_l |
| Phenotype | group.cls#h\_versus\_l |
| Upregulated in class | l |
| GeneSet | KEGG\_EPITHELIAL\_CELL\_SIGNALING\_IN\_HELICOBACTER\_PYLORI\_INFECTION |
| Enrichment Score (ES) | -0.44955918 |
| Normalized Enrichment Score (NES) | -1.5873183 |
| Nominal p-value | 0.052529182 |
| FDR q-value | 0.8686381 |
| FWER p-Value | 0.632 |
Table: GSEA Results Summary

  

Fig 1: Enrichment plot: KEGG\_EPITHELIAL\_CELL\_SIGNALING\_IN\_HELICOBACTER\_PYLORI\_INFECTION      
 Profile of the Running ES Score & Positions of GeneSet Members on the Rank Ordered List

  

| SYMBOL | TITLE | RANK IN GENE LIST | RANK METRIC SCORE | RUNNING ES | CORE ENRICHMENT || 1 | CXCR2 | na | 3338 | 0.238 | -0.0572 | No |
| 2 | ATP6V0D2 | na | 4031 | 0.213 | -0.0423 | No |
| 3 | ATP6V1B2 | na | 9429 | 0.079 | -0.1850 | No |
| 4 | MAPK10 | na | 10497 | 0.065 | -0.2052 | No |
| 5 | MAPK11 | na | 10512 | 0.064 | -0.1950 | No |
| 6 | ATP6V0A4 | na | 11197 | 0.056 | -0.2056 | No |
| 7 | MAPK9 | na | 11537 | 0.052 | -0.2068 | No |
| 8 | TJP1 | na | 11569 | 0.052 | -0.1993 | No |
| 9 | CSK | na | 11685 | 0.050 | -0.1944 | No |
| 10 | MAPK13 | na | 11811 | 0.049 | -0.1899 | No |
| 11 | ATP6V0B | na | 11849 | 0.049 | -0.1830 | No |
| 12 | PLCG1 | na | 11955 | 0.048 | -0.1783 | No |
| 13 | ATP6V0E1 | na | 12510 | 0.042 | -0.1873 | No |
| 14 | CASP3 | na | 13339 | 0.035 | -0.2054 | No |
| 15 | ATP6AP1 | na | 14609 | 0.026 | -0.2378 | No |
| 16 | RAC1 | na | 14759 | 0.025 | -0.2380 | No |
| 17 | ATP6V0E2 | na | 14809 | 0.024 | -0.2355 | No |
| 18 | NOD1 | na | 15698 | 0.018 | -0.2582 | No |
| 19 | ATP6V1G3 | na | 15776 | 0.017 | -0.2575 | No |
| 20 | GIT1 | na | 16349 | 0.014 | -0.2718 | No |
| 21 | ATP6V0D1 | na | 16641 | 0.012 | -0.2783 | No |
| 22 | ATP6V1F | na | 16959 | 0.010 | -0.2859 | No |
| 23 | MAPK14 | na | 17505 | 0.006 | -0.3006 | No |
| 24 | ATP6V1C1 | na | 17751 | 0.005 | -0.3069 | No |
| 25 | ATP6V0A1 | na | 18461 | -0.001 | -0.3272 | No |
| 26 | MAP2K4 | na | 18667 | -0.002 | -0.3328 | No |
| 27 | ATP6V1D | na | 18807 | -0.003 | -0.3363 | No |
| 28 | CHUK | na | 18953 | -0.004 | -0.3398 | No |
| 29 | ATP6V1A | na | 19008 | -0.005 | -0.3407 | No |
| 30 | PLCG2 | na | 19340 | -0.007 | -0.3490 | No |
| 31 | ATP6V1G1 | na | 19379 | -0.008 | -0.3489 | No |
| 32 | PTPN11 | na | 19596 | -0.009 | -0.3535 | No |
| 33 | ATP6V0C | na | 19779 | -0.011 | -0.3570 | No |
| 34 | ATP6V1E1 | na | 20236 | -0.015 | -0.3677 | No |
| 35 | ATP6V1C2 | na | 20822 | -0.020 | -0.3812 | No |
| 36 | F11R | na | 20999 | -0.022 | -0.3826 | No |
| 37 | ATP6V1H | na | 21127 | -0.023 | -0.3825 | No |
| 38 | EGFR | na | 22041 | -0.033 | -0.4035 | No |
| 39 | IKBKB | na | 22300 | -0.035 | -0.4052 | No |
| 40 | MAPK8 | na | 22620 | -0.039 | -0.4080 | No |
| 41 | CDC42 | na | 22626 | -0.039 | -0.4018 | No |
| 42 | IKBKG | na | 23063 | -0.044 | -0.4072 | No |
| 43 | JUN | na | 24077 | -0.057 | -0.4270 | No |
| 44 | ATP6V1E2 | na | 24858 | -0.069 | -0.4383 | Yes |
| 45 | ADAM17 | na | 25152 | -0.073 | -0.4348 | Yes |
| 46 | MAPK12 | na | 25666 | -0.081 | -0.4362 | Yes |
| 47 | NFKB1 | na | 25842 | -0.084 | -0.4274 | Yes |
| 48 | HBEGF | na | 26124 | -0.090 | -0.4207 | Yes |
| 49 | ADAM10 | na | 26855 | -0.104 | -0.4246 | Yes |
| 50 | CXCL8 | na | 26911 | -0.106 | -0.4089 | Yes |
| 51 | NFKBIA | na | 27480 | -0.118 | -0.4058 | Yes |
| 52 | ATP6V1B1 | na | 27614 | -0.121 | -0.3897 | Yes |
| 53 | ATP6V1G2 | na | 27824 | -0.127 | -0.3749 | Yes |
| 54 | PAK1 | na | 28053 | -0.132 | -0.3598 | Yes |
| 55 | LYN | na | 28223 | -0.136 | -0.3424 | Yes |
| 56 | RELA | na | 28616 | -0.147 | -0.3296 | Yes |
| 57 | SRC | na | 28777 | -0.151 | -0.3095 | Yes |
| 58 | MET | na | 29439 | -0.170 | -0.3006 | Yes |
| 59 | PTPRZ1 | na | 31401 | -0.236 | -0.3184 | Yes |
| 60 | MAP3K14 | na | 32153 | -0.273 | -0.2954 | Yes |
| 61 | CCL5 | na | 32225 | -0.277 | -0.2519 | Yes |
| 62 | JAM2 | na | 32852 | -0.317 | -0.2179 | Yes |
| 63 | CXCL1 | na | 33274 | -0.350 | -0.1726 | Yes |
| 64 | CXCR1 | na | 33350 | -0.357 | -0.1161 | Yes |
| 65 | IGSF5 | na | 33408 | -0.362 | -0.0584 | Yes |
| 66 | JAM3 | na | 34561 | -0.588 | 0.0048 | Yes |
Table: GSEA details [plain text format]

  

Fig 2: KEGG\_EPITHELIAL\_CELL\_SIGNALING\_IN\_HELICOBACTER\_PYLORI\_INFECTION      
 Blue-Pink O' Gram in the Space of the Analyzed GeneSet

  

Fig 3: KEGG\_EPITHELIAL\_CELL\_SIGNALING\_IN\_HELICOBACTER\_PYLORI\_INFECTION: Random ES distribution      
 Gene set null distribution of ES for **KEGG\_EPITHELIAL\_CELL\_SIGNALING\_IN\_HELICOBACTER\_PYLORI\_INFECTION**
